# Immune Checkpoint Inhibitors in Triple Negative Breast Cancer Treatment: Promising Future Prospects

**DOI:** 10.3389/fonc.2020.600573

**Published:** 2021-02-25

**Authors:** Remy Thomas, Ghaneya Al-Khadairi, Julie Decock

**Affiliations:** ^1^ Cancer Research Center, Qatar Biomedical Research Institute (QBRI), Hamad Bin Khalifa University (HBKU), Qatar Foundation (QF), Doha, Qatar; ^2^ College of Health and Life Sciences (CHLS), Hamad Bin Khalifa University (HBKU), Qatar Foundation (QF), Doha, Qatar

**Keywords:** triple negative breast cancer, immune checkpoint blockade, predictive biomarkers, tumor mutational burden, tumor infiltrating lymphocytes, combination therapy, programmed death-1 (PD-1), programmed death ligand-1 (PD-L1)

## Abstract

Immunotherapy has emerged as the fifth pillar of cancer treatment alongside surgery, radiotherapy, chemotherapy, and targeted therapy. Immune checkpoint inhibitors are the current superheroes of immunotherapy, unleashing a patient’s own immune cells to kill tumors and revolutionizing cancer treatment in a variety of cancers. Although breast cancer was historically believed to be immunologically silent, treatment with immune checkpoint inhibitors has been shown to induce modest responses in metastatic breast cancer. Given the inherent heterogeneity of breast tumors, this raised the question whether certain breast tumors might benefit more from immune-based interventions and which cancer cell-intrinsic and/or microenvironmental factors define the likelihood of inducing a potent and durable anti-tumor immune response. In this review, we will focus on triple negative breast cancer as immunogenic breast cancer subtype, and specifically discuss the relevance of tumor mutational burden, the plethora and diversity of tumor infiltrating immune cells in addition to the immunoscore, the presence of immune checkpoint expression, and the microbiome in defining immune checkpoint blockade response. We will highlight the current immune checkpoint inhibitor treatment options, either as monotherapy or in combination with standard-of-care treatment modalities such as chemotherapy and targeted therapy. In addition, we will look into the potential of immunotherapy-based combination strategies using immune checkpoint inhibitors to enhance both innate and adaptive immune responses, or to establish a more immune favorable environment for cancer vaccines. Finally, the review will address the need for unambiguous predictive biomarkers as one of the main challenges of immune checkpoint blockade. To conclude, the potential of immune checkpoint blockade for triple negative breast cancer treatment could be enhanced by exploration of aforementioned factors and treatment strategies thereby providing promising future prospects.

## Introduction

Breast cancer constitutes a major health problem worldwide, accounting for 30% of all female cancer cases and 15% of female cancer-related deaths (1). Clinically, breast tumors are categorized into hormone receptor positive (HR+) tumors expressing the estrogen (ER) and/or progesterone (PR) receptors, Human Epidermal Receptor 2 (Her2)-enriched tumors with overexpression of Her2 in the absence of HR expression, and triple negative tumors lacking expression of all three receptors. Standard treatment of these clinical subtypes consists of surgery, radiotherapy, chemotherapy, hormonal therapy, anti-Her2 targeted therapy or a combination thereof. In recent years, with -omics based profiling becoming more accessible and affordable, molecular profiling of tumors has started to enter clinical routine such as the multigene OncotypeDX, Mammaprint and ProSigna tests (2–4). Each of these assays uses distinct gene signatures to predict the risk of recurrence of early stage, hormone receptor positive (and negative) breast cancer. In addition, the OncotypeDX test helps to predict the likely benefit of adjuvant chemotherapy in early stage HR+ cancer. The more recent Prosigna test not only provides a 10-year risk of recurrence score but also classifies breast tumors into distinct prognostic molecular subtypes based on the Prediction Analysis of Microarray 50 (PAM50) gene signature. This signature forms the basis of the PAM50 classifier that has provided major insights into the molecular heterogeneity of breast tumors (5, 6). More specifically, the classifier categorizes breast tumors into four distinct molecular subtypes with different response to treatment and clinical outcome: luminal A (LA), luminal B (LB), Her2-enriched (Her2+), and basal-like (BL). Furthermore, stratification of breast tumors based on the presence of tumor infiltrating lymphocytes (TILs) and differential expression of immune-related genes revealed further heterogeneity with prognostic significance (6–9). Using a gene signature composed of immune-regulatory genes, chemokine ligands and genes involved in T helper 1 (Th1) signaling and effector immune functions, approximately 30% of basal-like and Her2-enriched breast tumors can be classified as tumors with an immune favorable phenotype as compared to 5–10% of luminal type tumors (9). In this review, we will look into cancer cell-intrinsic and/or microenvironmental factors that have a likely effect on shaping the tumor immune phenotype, and will discuss the emerging potential of immune checkpoint inhibitors (ICIs) in triple negative breast cancer treatment in particular.

## Potential of Immunotherapy in (Triple Negative) Breast Cancer: Parameters to be Considered

Cancer immunotherapy is considered the new pillar of cancer treatment, shifting the focus from the tumor to the tumor microenvironment and was awarded the Nobel Prize for physiology or medicine in 2018. Numerous immunotherapy approaches have proven effective in generating durable clinical responses, with the greatest success stories to date coming from treatment with immune checkpoint inhibitors (10–13). It is well known that tumors adopt various mechanisms to evade detection and eradication by the immune system, including the activation of inhibitory pathways governed by immune checkpoints. Treatment with ICIs releases the immune system from these inhibitory signals and reinvigorates the anti-tumor immune response as demonstrated by numerous studies and clinical trials using monoclonal antibodies against cytotoxic T-lymphocyte associated antigen-4 (CTLA-4), programmed death-1 (PD-1), and programmed death ligand-1 (PD-L1) (14–18). In breast cancer, especially triple negative breast cancer, treatment with ICIs has been found to improve clinical outcome (18). Overall, immune checkpoint inhibition is well tolerated and is associated with a relatively mild toxicity profile. However, immune-related adverse events may develop and need to be closely monitored, including the development of colitis, thyroid dysfunction, hypophysitis, skin rash, pneumonitis, and inflammatory arthritis (19).

The success of immunotherapy largely depends on the immunogenic nature of the tumor, exemplified by the higher response rates in malignant melanoma and non-small cell lung carcinoma (20, 21). Traditionally, breast cancer has been considered an immune silent cancer type that is less likely to benefit from immunotherapy. Increasing evidence, however, indicates that breast cancer constitutes a varied spectrum of tumors with different degrees of immunogenicity whereby triple negative breast cancer is believed to be a more immunogenic subtype (7–9, 22, 23). Moreover, multiple factors derived from tumor cells or from within the tumor micro- or macro-environment dictate the immune contexture of a tumor and hence responsiveness to immunotherapy, including the tumor mutational burden (TMB) and neoantigen load, diversity of the immune infiltrate and the microbiome.

### Tumor Mutational Burden and Neoantigen Load

The tumor mutational burden is defined as the total number of somatic nonsynonymous mutations in the coding region of genes that may result in the generation of abnormal proteins or neoantigens (24, 25). A high TMB and number of predicted neoantigens has been associated with a better response to immune checkpoint inhibitor therapy in various cancer types (26–30). In breast cancer, most tumors harbor a low TMB (1mut/Mb) and only 5% of all tumors are characterized by a high tumor mutational burden (≥ 10 mut/Mb) of which most are metastatic (31, 32). More specifically, TNBCs have a higher TMB compared to Her2-enriched and HR+ tumors (33, 34). Analysis of the TCGA and METABRIC breast cancer datasets demonstrate an improved overall survival (OS) for patients with tumors featuring a high TMB and favorable immune-infiltrate disposition (FID), irrespective of the type of treatment. Luminal A tumors with a high TMB/FID phenotype were associated with the best survival rates, whereas TNBCs with a high TMB and poor immune-infiltrate disposition were associated with the worst prognosis (25). Conversely, immune-rich TNBC tumors with lower mutation and neoantigen counts have been associated with better prognosis, likely due to a reduced clonal heterogeneity as a result of immunosurveillance (35).

Furthermore, tumors with somatic or germline *BRCA1/2* mutations are believed to be more immunogenic due to the dysregulation of homologous recombination-based DNA repair, leading to increased genomic instability and higher mutational burden (36). However, *BRCA1/2* mutation-associated breast tumors display a great variability in immunogenicity with approximately 50% of tumors displaying an absent or mild tumor lymphocyte infiltrate and moderate neoantigen load, suggesting that only a subset of *BRCA1/2* breast tumors may benefit from immune-based therapy (37). In line with this, at best 1 out of 5 patients with triple negative breast cancer, the most common form of *BRCA1* mutation-associated breast cancer, has been shown to benefit from single agent PD-1 blockade (38–40). Interestingly, genomic analysis of 115 *BRCA1/2* breast tumors revealed an inverse association between homologous recombination deficiency (HRD) and immunogenicity despite a higher mutational burden and neoantigen load (41). Moreover, hormone receptor status further stratified *BRCA1/2* breast tumors with low-HRD TNBC tumors being more immunogenic than high-HRD HR+ tumors (41). This unexpected inverse correlation of high TMB, resulting from homologous recombination deficiency, and immunogenicity is supported by a pan-cancer analysis that demonstrated that large somatic copy number alterations are associated with reduced immunogenicity, possibly due to disruption of genes involved in the regulation of immune cell recruitment (42). In accordance, PTEN, another important regulator of DNA damage repair and hence mutational burden, is frequently impaired in tumors and loss of PTEN has been associated with poor response to PD-1 blockade (43, 44). For instance, patients with metastatic TNBC (mTNBC) who carry *PTEN* mutations had a significant lower response rate to PD-1/PD-L1 inhibitors (45). Moreover, in the absence of PTEN-mediated inhibition of the PI3K-Akt pathway, the use of an Akt inhibitor combined with chemotherapy and PD-L1 blockade significantly improved the overall response rate of metastatic TNBC patients compared to combination treatments of chemotherapy with PD-L1 blockade or Akt inhibition (46). Together, these findings suggest that in a proportion of breast tumors ICI response is not dictated by TMB per se but rather by specific genomic events that disrupt a functional immune response.

### Diversity of Immune Infiltrate

In addition to cancer cell-intrinsic features, the tumor microenvironment plays a prominent role in determining anti-tumor immunity and response to immunotherapy. Understanding the complexity of the interplay between tumor cells and components of the immune system offers a unique opportunity to explore combination treatments that can help to reshape the tumor microenvironment into an immune favorable phenotype. Immunohistochemical analyses of tumor immune infiltrates has resulted in the classification of tumors into distinct immune phenotypes: “hot”, “cold-immune desert”, and “cold-excluded” tumors (47–49). Immunological “hot” tumors often have a high TMB and number of neoantigens, and have a high likelihood of provoking an anti-tumor immune response. They are also called “inflamed tumors” as they are characterized by a considerable infiltration of T cells although these are not fully functional. Overall, hot tumors are associated with a better response to ICIs through the activation of the present immune infiltrate (50) and examples include melanoma, non-small cell lung cancer, head and neck cancer, kidney, liver, and bladder cancer. Immunological “cold” tumors either exhibit a lack or paucity of a T cell infiltrate, the so-called “immune desert” tumors, or feature a phenotype whereby T cells have been excluded from the tumor core and aggregate at the tumor boundaries, the so-called “immune excluded” tumors. Tumors with an “immune excluded” phenotype reflect the ability to induce a T cell- mediated immune response, however, the response is impaired by the inability to penetrate the tumor tissue. The presence of immunosuppressive immune cell subsets within the tumor or tumor microenvironment can alter both the infiltration and functional status of the T cell infiltrate and hence reduce the potential benefit from ICI therapy (48). Many studies are looking into ways to turn “cold” tumors into “hot” tumors to achieve higher responsiveness to immune checkpoint blockade. Here, we will discuss some of the factors to be considered in addition to the density and localization of the immune infiltrate such as the cellular composition and functional orientation of the immune cell infiltrate and of tertiary lymphoid structures (TLS), the expression of immune checkpoints, and the enrichment of prognostic immune gene signatures.

Tumor infiltrating lymphocytes or TILs represent the major infiltrating immune cell subpopulation defining a favorable immune microenvironment in tumors. The density of TILs is indicative of the magnitude of anti-tumor immunity and is emerging as a prognostic and predictive biomarker for immunotherapy response in a wide range of cancers (51–53). The seminal work by Galon et al. introduced the immunoscore concept in colorectal cancer, an immunohistochemically-based scoring system of CD8+ TILs in the center and invasive margin of a tumor with independent prognostic connotation (47). Subsequent work consolidated the prognostic value of the immunoscore in colorectal cancer and multiple other cancers (53–56). A recent study on the predictive value of the immunoscore in colorectal cancer patients suggests that patients with a low immunoscore do not benefit from a longer treatment with oxaliplatin-based chemotherapy as opposed to patients with intermediate or high immunoscore values (55). This observation seems counterintuitive as one could argue that patients with low immunoscore and higher risk of recurrence would more likely benefit from longer treatment. However, it is important to consider the interactions of the chemotherapeutic agents with the immune response. Oxaliplatin is known to elicit bona fide immunogenic cell death and 5-fluorouracil decreases the number of myeloid derived suppressor cells (MDSCs) while enhancing the cytotoxic T cell function, however, these effects depend on the presence of an active tumor immune microenvironment. Therefore, tumors with a low immunoscore and weak cytotoxic T cell activity may not experience additional benefit from increasing the treatment duration. More studies are needed to validate these findings as the follow-up time of the current study was rather short with 4.3 years. In breast cancer, the immunoscore has not yet been established as a prognostic and/or predictive biomarker, however, a plethora of studies supports the importance of the tumor immune microenvironment in defining breast cancer clinical outcome. Numerous studies have demonstrated an association of breast tumor infiltration by cytotoxic T lymphocytes with better survival (57–60). In particular, TNBC and Her2-enriched tumors feature high TIL counts, which are associated with better clinical outcome, and suggest greater immunogenicity and likely benefit from immune-based interventions (61, 62). Higher densities of TILs have also been associated with greater response rates to chemotherapy (62–65).

In line with the immunoscore concept, spatial distribution of lymphocytes beyond intratumoral lymphocytes could provide added value for predicting survival and treatment response in breast cancer. High densities of stromal T lymphocytes have been associated with improved breast cancer specific survival of patients with TNBC and Her2-enriched tumors (66). Moreover, one study expanded the immunoscore concept by quantifying the density of immunosuppressive FoxP3 T regulatory cells (Treg) in addition to CD3+ and CD8+ T cells (67). Interestingly, they were able to develop a prognostic scoring system that could distinguish molecular breast cancer subtypes. Joint analysis of immunosuppressive CD163+ tumor associated macrophages (TAMs) with cytotoxic CD8+ T lymphocytes resulted in a novel immune infiltrate scoring model with favorable prognosis, as defined by high CD8+ and low CD163+ cell counts in the tumor center and low CD8+ and high CD163+ in the invasive tumor margin (68, 69). These findings highlight the importance of capturing a complete picture of the tumor immune microenvironment, accounting for both cytotoxic T cells and immunosuppressive immune cell populations. This notion is further supported by the ongoing discussion on the prognostic value of tertiary lymphoid structures within the tumor or tumor microenvironment. Several studies in a range of cancer types have reported a favorable outcome for patients with a high number of TLS, irrespective or in addition to a high TIL count (70, 71). In TNBC, high TIL counts in combination with moderate to high TLS counts have been associated with improved disease free survival (DFS) (70). On the other hand, a number of studies have reported conflicting data that do not support a favorable prognostic value for TLS (69). Notably, TLS can exert a dual effect on anti-tumor immunity, serving as an *in situ* niche of cytotoxic T cells as well as of immunosuppressive cells such as T regulatory cells and hence high TLS counts can be associated with better or worse prognosis (72). High TLS counts have been associated with better DFS in patients with Her2-enriched tumors whereas no prognostic value was observed in Her2-negative breast cancer patients. Therefore, it is clear that the current definition of the tumor immune microenvironment needs to be revisited in order to account for TLS cellular composition and functional orientation.

This brings us to the pivotal role of the activation status of the tumor immune infiltrate which is partly controlled by the expression of immune checkpoints. The presence of infiltrating T lymphocytes has been associated with elevated expression of PD-L1 (73–75), corroborating the therapeutic potential of immune checkpoint blockade in tumors with a high T cell immune infiltrate density. In accordance, high TIL scores in patients with TNBC and Her2-enriched tumors predict a better response to PD-1 inhibitors, counteracting the increased PD-L1 expression (51, 76, 77). In a study involving more than 3,000 breast cancer patients, the relevance of TILs for chemotherapy response and prognosis in patients of different breast cancer subtypes was assessed (78). Increased TIL counts were associated with a survival benefit and better response to neoadjuvant chemotherapy in Her2-enriched breast cancer and TNBC. In contrast, a different role for TILs was observed in luminal breast cancer where an increase in TILs was associated with adverse prognostic effects. Furthermore, combined analysis of TIL density and PD-L1 tumor expression indicated that the DFS of TNBC patients with low-TIL tumors (< 30% stromal) was significantly worse compared to patients with high-TIL tumors, with the most unfavorable DFS and OS for patients with low-TIL and high PD-L1 (> 50%) (75). Furthermore, the presence of specifically tissue resident memory T cells in the TIL infiltrate of TNBC tumors has been associated with better response rates and overall survival in patients who received chemotherapy or PD-1 inhibition (51, 79, 80). Interestingly, characterization of TILs after treatment with PD-1/PD-L1 inhibitors revealed an increase in expression of various immune checkpoints including PD-1, CTLA-4, T cell immunoglobulin and mucin domain-containing protein 3 (Tim3) and Lymphocyte-activation gene 3 (Lag3) in CD4+ T cell subsets suggesting the presence of a compensatory inhibitory mechanism mediated by CD4+ T regulatory cells (81). These findings underscore the need to identify, quantify, and phenotype all components of the immune microenvironment including immunosuppressive regulators. Great efforts are expended to develop strategies to deplete immunosuppressive cells from the tumor microenvironment, to impede their infiltration and to impair their functionality, or to induce cytotoxic T cell expansion, survival and function by modulating cytokine levels (82–84). Importantly, any of these strategies could be combined with immune checkpoint inhibitors. In this context, it is important to note that PD-1 and CTLA-4 are not only expressed on activated T cells, but also on T regulatory cells. Hence, treatment with anti-PD-1 and/or anti-CTLA4 antibodies may result in the additional release of Treg-mediated suppression of T cell activation, strengthening the anti-tumor immunity (85–88). Of note, additional factors besides immune checkpoint expression probably affect ICI response and clinical outcome as for instance, only a small proportion of metastatic PD-L1 positive TNBC patients (8–20%) respond to PD1/PD-L1 therapy (76).

In an attempt to comprehensively capture the immune contexture of a tumor, numerous immune gene signatures have been developed. The first prognostic immune signature describing the functional orientation of the tumor immune microenvironment was established in colorectal cancer and was composed of genes involved in Th1 and cytotoxic T cell function, including interferon- γ (IFN-γ), granulysin (GNLY), perforin (PRF1), and granzymes (GZMs) (47). This signature was subsequently validated in other cancer types including breast cancer (89, 90). In addition, Hendrickx et al. demonstrated that the Immunologic Constant of Rejection (ICR) 20-gene signature can differentiate immune favorable and immune unfavorable breast cancer subtypes, and recently refined and validated its prognostic value in a pan-cancer study (9, 91) Furthermore, they showed that MAPK pathway regulation could modulate the intratumoral response in breast cancer (9). A meta-analysis of approximately 18,000 human tumors identified complex associations between 22 distinct leukocyte subsets and cancer survival (92). Using the CIBERSORT algorithm for relative immune cell abundance, the authors demonstrated that tumor-associated neutrophil and plasma cell signatures are significant but opposite predictors of survival in breast cancer. Further, a T cell- inflamed gene expression profile exhibited predictive value to identify pan-cancer patients that will more likely benefit from PD-1 inhibition (93). Paradoxically, subsets of breast cancer patients with high expression of immune-associated signatures have been identified to experience poor outcome (94), suggesting the presence of additional complexity beyond the current information provided by bulk tumor immune signatures. Moreover, some studies have demonstrated differences in spatial distribution of immune gene signatures (95). For instance, integration of CD8+ T cell localization and matched stromal and epithelial tumor gene expression signatures revealed distinct, spatial, tumor immune microenvironment-subtypes of treatment-naive TNBC tumors, each characterized by a specific metagene signature (96).

### Gut and Breast Microbiome

The gut microbiome is a recognized master modulator of the development and maintenance of a healthy immune system (97, 98). Perturbation of the normal microbiota—dysbiosis—is often observed in disease and changes the interactions between the gut microbiota, intestinal epithelium, and host immune system (99). Many studies have shown that gut microbiota shape the immune system and the host metabolism. In addition to regulating local, intestinal immune responses, changes in gut microbiota can have systemic effects on the innate and adaptive immunity. While the encounter of microbial molecules by Toll-like receptors provoke a local immune response in the gut, the escape of microbial factors from the gut can modulate immune function, causing systemic infection or inflammation which favors the development of immune-mediated and metabolic diseases (100). Thus, understanding how the gut microbiota impact anti-tumor immunity could provide insight into how it might influence tumor development, progression and treatment response. In breast cancer, a collection of microbial genes known as the estrobolome has been shown to affect estrogen metabolism, resulting in higher circulating levels of estrogen and hence an increased risk of hormone-dependent breast cancer (101). Furthermore, the gut microbiome has been found to be involved in the regulation of tumor progression and the response to anticancer therapies (102–106). For instance, gut microbiome dysbiosis has been shown to promote cancer cell dissemination in a HR+ breast cancer mouse model through increased fibrosis and collagen deposition (107). Several studies have identified distinct microbial signatures in breast cancer patients, however, further studies are needed to define their diagnostic and therapeutic implications (108–110). Furthermore, few studies demonstrated that the composition of the gut microbiota could influence the response to immunotherapy, including immune checkpoint. For instance, comparing the gut and oral microbiome of melanoma patients treated with anti-PD-1 immunotherapy revealed significant differences in the diversity and composition of gut microbiota in patients that responded to treatment versus non-responders (103, 105). Furthermore, exposure to broad-spectrum combination antibiotics (fluoroquinolones, ß-lactam^+/-^ or macrolides) during anti-PD1/PD-L1 treatment has been shown to significantly decrease progression-free survival (PFS) and OS of patients with advanced non-small cell lung, renal cell carcinoma, and urothelial carcinoma, suggesting that the overall diversity of the microbiota and the presence of specific clades determines the responsiveness to immunotherapy (104).

Historically, breast tumor tissue has been considered a sterile environment, however, recent studies suggest the existence of a local, breast microbiome. Indeed, the composition of breast tissue with abundance of fatty tissue, extensive vasculature, and lymphatic drainage makes it a favorable environment for the growth of bacteria (111). Comparison of microbial signatures across multiple cancer types revealed cancer type specific microbial signatures that differ between the respective tumors and adjacent normal tissues whereby breast cancer was associated with a particularly rich and diverse microbiome. Furthermore, the breast microbiome has been shown to differ from normal to benign to malignant tissues, as well as between breast cancer subtypes, and in relation to response to immunotherapy (112, 113).

## Immune Checkpoint Inhibition in Triple Negative Breast Cancer

**Figure 1:**
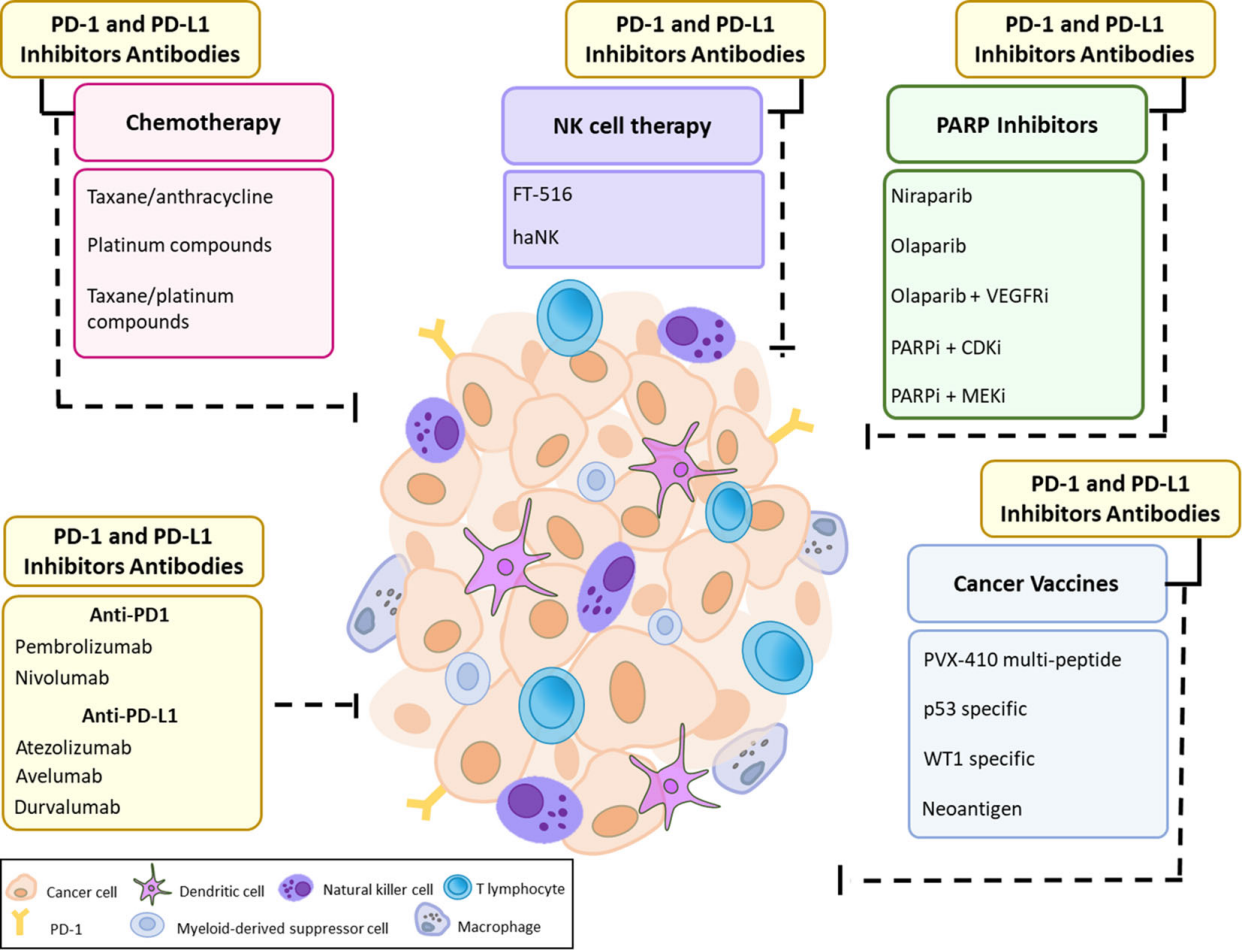
Current Approaches for PD-1 and PD-L1 immune checkpoint inhibition in TNBC. The efficacy of PD-1 and PD-L1 therapy may be hampered due to cancer cell-intrinsic interactions and/or microenvironmental factors along with the expression of immune checkpoint molecules such as PD-L1 that define a potent and durable anti-tumor immune response. Immune checkpoint blockade could be used as monotherapy or in combination with different therapeutic approaches, including chemotherapy, PARP inhibitors with or without VEGFR/CDK/MEK inhibitors, cancer vaccines, and NK cell therapy.

ICI therapy has become the most successful immune-based intervention to generate durable responses in a variety of tumors. Monoclonal antibodies against PD-1/PD-L1 and CTLA-4 have emerged as powerful tools to release the inhibitory regulation of T cell activation (114, 115). To date, multiple blocking monoclonal antibodies have been approved by the US Food and Drug Administration (FDA) including the anti CTLA-4 antibody ipilimumab, anti-PD1 antibodies pembrolizumab, nivolumab and cemiplimab and anti-PD-L1 antibodies atezolizumab, avelumab and durvalumab (116, 117). Treatment response to immune checkpoint inhibitors varies greatly with only a small proportion of patients experiencing better survival rates (118, 119). Hence, there is a growing need for predictive biomarkers of ICI response. Furthermore, few preclinical studies are investigating the benefit of targeting multiple immune checkpoints including PD-1, CTLA-4, Tim3, and Lag3 (120). Currently, the majority of breast cancer studies focus on inhibition of the PD1/PD-L1 pathway. A single-arm pilot study investigating the combination of PD1/PD-L1 blockade with CTLA-4 inhibition reported an objective response rate (ORR) of 43% in patients with metastatic TNBC, whereas no responses were observed in patients with HR+ breast cancer (121). We will focus our discussion on anti-PD1/PD-L1 mono- and combination therapy in TNBC (**Figure 1**) given that it is the most immunogenic breast cancer subtype and hence will more likely benefit from treatment with ICIs.

### PD1/PD-L1 Antibody Monotherapy

PD1/PD-L1 monotherapy has demonstrated promising durable responses in patients with advanced, metastatic TNBC (**Table 1**). The safety profile and clinical activity of the anti-PD1 inhibitor pembrolizumab was first studied in heavily pretreated patients with advanced, PD-L1 positive triple negative breast cancer, head and neck cancer, urothelial cancer or gastric cancer in the KEYNOTE-012 (NCT01848834) clinical trial. Interim analysis revealed an overall response rate of 18.5% in mTNBC patients with the median duration of response ranging from 15.0 to 47.3 weeks (38). In a subsequent phase II clinical trial, KEYNOTE-086 (NCT02447003), PD-L1 positive mTNBC patients who received no prior systemic treatment for metastatic disease showed the highest ORR of 21.4% with a median duration of response of 10.4 months at data cut-off, and PFS and OS of 2.1 and 18.0 months, respectively (80). In comparison, heavily pretreated, PD-L1 positive mTNBC patients experienced an ORR of 5.7% with median PFS and OS of 2.0 and 9.0 months, respectively (122). Both studies demonstrated a manageable safety profile and durable clinical activity of single agent pembrolizumab treatment in PD-L1 positive mTNBC, in particular in the first-line setting. Next, the randomized phase 3 KEYNOTE-119 trial (NCT02555657) investigated the efficacy of pembrolizumab monotherapy versus chemotherapy (capecitabine, gemcitabine, eribulin, vinorelbine) in pretreated, PD-L1 positive mTNBC. Initial results revealed no significant improvement in PFS (HR = 1.35) nor in OS (HR = 0.86) for patients receiving pembrolizumab, although there was a trend for better survival with higher PD-L1 score (123). At the date of data cut-off (11^th^ April 2019), the median follow-up time was 9.9 months for the pembrolizumab cohort and 10.9 months for the chemotherapy cohort, hence, differential survival outcomes may become more apparent as the study matures. However, these findings may also suggest that pembrolizumab monotherapy is more effective as first line treatment in mTNBC.

**Table 1:** PD1/PD-L1 antibody monotherapy in metastatic TNBC.

NCT Number	Other IDs	Intervention	Trial status/interim results	Ref
**NCT01848834**	KEYNOTE-012/MK-3475-012, 2012-005771-14, 142453, 3475-012	pembrolizumab	completed ORR 18.5%	(38)
**NCT02447003**	KEYNOTE-086/MK-3475-012, 3475-086, 2015-000294-13, 152987	pembrolizumab	completed ORR 5.7% PFS 2.0 mths, OS 9.0 mths first line setting: ORR 21.4% PFS 2.1 mths, OS 18.0 mths	(80, 122)
**NCT02555657**	KEYNOTE-119/MK-3475-119, 3475-119, 2015-001020-27, 153082	pembrolizumab vs chemotherapy	active no difference in PFS and OS	(123)
**NCT01375842**	PCD4989g, 2011-001422-23, GO27831	atezolizumab	completed ORR 6% (12 vs 0%^*^) OS (10.1 vs 6.0 mths^*^) first line setting: ORR 24% OS 17.6 mths	(124)
**NCT01772004**	JAVELIN/EMR 100070-001, 2013-002834-19	avelumab	completed ORR 5.2% (22.2 vs 2.6%^**^)	(40)
**NCT02926196**	A-Brave, 2016-000189-45	avelumab	recruiting	

ORR, overall response rate; OS, overall survival; PFS progression free survival.

*PD-L1 cutoff 1%; **PD-L1 cutoff 10%.

In addition to blocking PD-1, antibodies have been developed that target PD-L1, thereby disrupting PD-L1/CD80 binding in addition to PD-L1/PD1 and resulting in an augmented anti-tumor immune response by both T cells and antigen presenting cells (125). In breast cancer, studies have investigated the safety profiles and efficacy of two anti PD-L1 antibodies, atezolizumab and avelumab. The clinical activity of single agent atezolizumab treatment was evaluated in a multi-cohort phase I study (NCT01375842) involving patients with locally advanced or metastatic solid malignancies or hematologic malignancies. In mTNBC, the ORR in first line atezolizumab treatment reached 24% with a median OS of 17.6 months compared to 6% in pretreated patients (124). PD-L1 expression in at least 1% of tumor infiltrating immune cells was associated with higher ORR (12 versus 0%) and better OS (10.1 versus 6.0 months). Further, higher levels of PD-L1 positivity (> 10%) were associated with better ORR and OS, albeit not significantly. The phase 1b JAVELIN trial (NCT01772004) on avelumab reported an ORR of 3.0% in metastatic breast cancer, and an ORR of 5.2% in mTNBC (40). In line with previous reports, higher response rates were observed in PD-L1 positive versus negative patients (16.7 vs 1.6%) using a PD-L1 cutoff of 10%, in particular in TNBC patients (22.2 vs 2.6%). To conclude, although the response rates of single agent ICIs in mTNBC may be modest, the durable responses of a subset of PD-L1 positive patients suggest that combination treatment of immune checkpoint blockade with other treatment modalities may provide a favorable outcome.

### PD1/PD-L1 Antibody-Chemotherapy Combination Treatment

Chemotherapy has been shown to increase tumor cell antigen release, induce the expression of MHC Class I molecules, neoantigens and PD-L1, and promote dendritic cell activation thus potentially augmenting the released immune response following or during ICI treatment (126–128). In line with this rationale, combination regimens of PD1/PD-L1 inhibitors with chemotherapy have shown promising results in metastatic, locally advanced and early stage TNBC (**Table 2**).

**Table 2:** PD1/PD-L1 antibody chemotherapy combination treatment in TNBC.

NCT Number	Other IDs	Intervention	Disease setting	Trial status/interim results	Ref
**NCT02819518**	KEYNOTE-355/MK-3475-355, 3475-355, 2016-001432-35, 163422	pembrolizumab + nab-paclitaxel or paclitaxel or gemcitabine/carboplatin	metastatic	active first line setting: PFS 9.7 mths	(129)
**NCT02755272**	BR-076	pembrolizumab + gemcitabine/carboplatin	metastatic	recruiting	
**NCT02513472**	KEYNOTE-150, ENHANCE 1, E7389-M001-218	pembrolizumab + eribulin mesylate	metastatic	active ORR 25.6% PFS 4.1 mths	(130)
**NCT02499367**	TONIC, N15TON	cyclophosphamide, cisplatin or doxorubicin followed by nivolumab	metastatic	active ORR 35% (doxorubicin) first line setting: ORR 17%	(117)
**NCT01042379**	I-SPY 2, 097517	neoadjuvant pembrolizumab + paclitaxel, followed by AC	locally advanced	recruiting	(131)
**NCT02622074**	KEYNOTE-173/MK-3475-173, 3475-173, 2015-002405-11	neoadjuvant pembrolizumab + chemotherapy combination (nab-paclitaxel, paclitaxel, doxorubicin, cyclophosphamide, carboplatin)	locally advanced	completed first line setting: pCR 60%	(132)
**NCT03036488**	KEYNOTE-522/MK-3475-522, 3475-522, 2016-004740-11, 173567	neoadjuvant pembrolizumab + paclitaxel-carboplatin followed by adjuvant pembrolizumab	locally advanced	active first line setting: pCR 64.8%	(133)
**NCT01633970**	GP28328, 2012-001422-10	atezolizumab + nab-paclitaxel	locally advanced, metastatic	active ORR 39.4% PFS 5.5 mths	(134)
**NCT02425891**	IMpassion130, WO29522, 2014-005490-37	atezolizumab + nab-paclitaxel	metastatic	active first line setting: ORR 53% OS 25 mths	(135)
**NCT03125902**	IMpassion131, MO39196, 2016-004024-29	atezolizumab + paclitaxel	locally advanced, metastatic	active first line setting	
**NCT03371017**	Impassion132, MO039193, 2016-005119-42	atezolizumab + gemcitabine/carboplatin or capecitabine	locally advanced, metastatic	recruiting	
**NCT02685059**	GeparNuevo, GBG89	neoadjuvant durvalumab + nab-paclitaxel + EC	early stage	unknown pCR 53%	(136)
**NCT02620280**	NeoTRIPaPDL1, FM-14-B02, 2014-005017-23	neoadjuvant atezolizumab + nab-paclitaxel + carboplatin, followed by AC or EC or FEC	early high risk, locally advanced	active	(137)
**NCT03197935**	Impassion031, WO39392, 2016-004734-22	neoadjuvant atezolizumab + nab-paclitaxel, followed by AC	early stage	active pCR 57.6%	(138)
**NCT03281954**	NSABP B-59/GBG 96-GeparDouze, 2017-002771-25, MO39875	neoadjuvant atezolizumab + paclitaxel + carboplatin, followed by adjuvant atezolizumab + AC or EC	early stage	recruiting	
**NCT03498716**	Impassion030, WO39391, 2016-003695-47, BIG 16-05, AFT-27, ALEXANDRA	atezolizumab + paclitaxel, followed by atezolizumab + AC or EC	locally advanced	recruiting	

AC, doxorubin + cyclophosphamide; EC, epirubicin + cyclophosphamide; FEC, fluorouracil + epirubicin + cyclophosphamide; ORR, overall response rate; OS, overall survival; pCR, pathological complete response; PFS, progression free survival.

The majority of studies on PD1 inhibition in TNBC has investigated the safety profile and clinical activity of pembrolizumab. Interim analysis of the phase 3 KEYNOTE-355 (NCT02819518) study reveals a significant improvement of PFS (5.6 vs 9.7 months) in strong PD-L1 positive, untreated mTNBC patients who received pembrolizumab in addition to chemotherapy (nab-paclitaxel, paclitaxel, gemcitabine/carboplatin) (129). Results from the phase 2 BR-076 (NCT02755272) clinical trial on pembrolizumab in combination with gemcitabine/carboplatin in mTNBC are pending. The KEYNOTE-150/ENHANCE 1 (NCT02513472) trial of pembrolizumab plus the microtubule inhibitor eribulin mesylate demonstrated an ORR of 25.6% with a median PFS of 4.1 months (130). The phase 2 TONIC trial (NCT02499367) evaluated the efficacy of PD1 blockade with nivolumab in pre-treated mTNBC (cyclophosphamide, cisplatin, doxorubicin). Of note, nivolumab therapy preceded by doxorubicin resulted in an ORR of 35 compared to 23% for cisplatin and 17% for patients without preceding chemotherapy, suggesting that pretreatment with chemotherapy can induce an inflamed tumor microenvironment (117). In comparison with metastatic TNBC, significant more studies have been conducted in locally advanced or early stage TNBC. In the phase 2 I-SPY 2 (NCT01042379) study the addition of pembrolizumab to taxane- and anthracycline-based neoadjuvant chemotherapy doubled the estimated pathological complete response (pCR) rates of early stage patients with Her2-negative breast cancer including triple negative breast cancer (131). These promising results provided the rationale for the phase 1 KEYNOTE-173 (NCT02622074) trial to investigate the toxicity and anti-tumor activity of adding pembrolizumab to six commonly used neoadjuvant chemotherapy regimens in untreated, locally advanced TNBC. The toxicity profile of the combination treatments were similar to what has been observed for the individual treatments, suggesting a manageable safety profile. Furthermore, combination treatment showed promising clinical activity with pCR rates of 60% across all treatment cohorts (132). In accordance with other studies, higher pre-treatment PD-L1 expression was associated with better outcome. Similarly, interim analysis of the phase 3 KEYNOTE-522 trial (NCT03036488) demonstrated that addition of pembrolizumab to paclitaxel-carboplatin chemotherapy in the neoadjuvant setting, followed by adjuvant pembrolizumab increased the pCR rates from 51.2 to 64.8% in untreated, locally advanced TNBC patients (133). Of note, the trial design does not allow the comparison of adjuvant pembrolizumab versus placebo treatment following neoadjuvant chemotherapy alone.

In addition to PD1 blockade, several clinical trials aim to study the safety and efficacy of PD-L1 inhibition in combination with chemotherapy, in particular in metastatic TNBC patients. The phase 1b clinical study NCT01633970 reported an ORR of 39.4% with a median PFS of 5.5 months for locally advanced or metastatic TNBC patients treated with atezolizumab plus nab-paclitaxel (134). PD-L1 positive mTNBC patients showed a non-significant higher ORR (41.4 vs 33.3%), PFS (6.9 vs 5.4 months) and OS (21.9 vs 11.4 months), irrespective of treatment history. Furthermore, although not statistically significant, patients who received the treatment regimen in first line setting experienced a higher ORR (53.8 vs 30.0%), longer PFS (8.6 vs 5.1 months) and OS (24.2 vs 12.4 months), providing evidence for a more favorable outcome compared to atezolizumab monotherapy where an ORR of 24% and median PFS of 1.6 months was observed (124, 134). The phase 3 randomized IMpassion130 trial (NCT02425891) supports these findings, demonstrating a clinically meaningful improvement in OS of 7 months (25.0 vs 18.0 months) for PD-L1 positive mTNBC patients who received first line atezolizumab plus nab-paclitaxel treatment (135). Interim results show that addition of pembrolizumab increased the ORR from 33 to 53% (128). In 2019, the FDA and European Medicines Agency (EMA) granted accelerated approval for the use of atezolizumab plus nab-paclitaxel as first line treatment of PD-L1-positive, unresectable, locally advanced or metastatic TNBC. The subsequent phase 3 IMpassion131 trial (NCT03125902) will evaluate the safety and efficacy of atezolizumab plus paclitaxel as a first-line therapy in patients with either locally advanced or metastatic TNBC. The IMpassion132 trial (NCT03371017) will investigate whether atezolizumab plus chemotherapy (gemcitabine/carboplatin, capecitabine) may benefit pretreated, inoperable locally advanced or metastatic TNBC patients who were not eligible for the IMpassion130 trial. So far, limited information is available on the effect of PD-L1 blockade in combination with chemotherapy for early stage TNBC. Results from the randomized phase 3 GeparNuevo study (NCT02685059) suggest that combining durvalumab with taxane-anthracycline based neoadjuvant chemotherapy provides clinical benefit in early TNBC with an increase in pCR from 44 to 53% (136). As of July 2020, no interim results are available for the phase 3 NeoTRIPaPDL1 (NCT02620280) clinical trial that aims to evaluate the anti-tumor activity of neoadjuvant atezolizumab plus carboplatin and nab-paclitaxel, followed by adjuvant chemotherapy in early stage high risk or locally advanced TNBC. Preliminary results were presented at the San Antonio Breast Cancer Symposium 2019 and revealed slightly higher pCR rates with pembrolizumab addition (137). The phase 3 NSABP B-59 (NCT03281954) trial of neoadjuvant chemotherapy (paclitaxel plus carboplatin) with atezolizumab, followed by adjuvant atezolizumab and chemotherapy is currently in the recruiting stage. A recent study released interim results from the Impassion031 (NCT03197935) trial on the combination treatment of neoadjuvant atezolizumab with sequential nab-paclitaxel and anthracycline-based chemotherapy in early stage TNBC. Patients who received atezolizumab plus chemotherapy showed a pathologic complete response rate of 57.6 versus 41.1% in patients who received chemotherapy plus placebo (138). In PD-L1 positive patients, the pathologic complete response reached 69% for patients who received atezolizumab plus chemotherapy and 49% for patients treated with chemotherapy plus placebo. Of note, there are two ongoing studies in locally advanced TNBC that evaluate the effect of chemotherapy with PD-L1 blockade in adjuvant setting. The Impassion30 (NCT03498716) trial will study the efficacy of atezolizumab in combination with adjuvant chemotherapy, while the A-Brave (NCT02926196) study focuses on avelumab.

### PD1/PD-L1 Antibody-Targeted Therapy Combination Treatment

Triple negative tumors feature a higher tumor mutational burden and extensive genomic instability with defects in the DNA damage response (139). As such, combination therapy strategies targeting distinct oncogenic pathways in conjunction with immunotherapy could offer a promising approach for TNBC treatment. The current clinical trials exploring such combination therapies are summarized in **Table 3**. For instance, Poly (ADP-Ribose) Polymerase inhibitors (PARPi) that target the homologous recombination repair pathway and induce synthetic lethality in *BRCA1/2* mutation carriers have been approved for the treatment of TNBC patients with germline mutations in *BRCA1/2* (143). The use of PARPi in combination with immune checkpoint blockade in this subset of TNBC patients has the potential to trigger a stronger anti-tumor immune response as a result of the activation of infiltrating T cells following the release of tumor antigens by PARPi-induced cell death. Furthermore, PARPi have been shown to upregulate PD-L1 expression in cell line and animal models providing further rationale for combining treatment with PD1/PD-L1 inhibitors (144). The KEYNOTE-162/TOPACIO (NCT02657889) study reported an ORR of 29% in mTNBC patients treated with a combination of pembrolizumab and the PARPi niraparib. The presence of *BRCA* mutations was associated with a higher ORR of 67% (140). Of note, the ORR was higher than what has been reported for anti-PD1 monotherapy in similar patient populations (122, 124). Additionally, several clinical trials have been designed to evaluate the combination of PD-L1 inhibition with PARPi in mTNBC, including two phase 2 studies combining durvalumab with the PARPi olaparib (DORA/NCT03167619 and NCT03801369), and a phase 2 study on atezolizumab plus olaparib (NCT02849496). Furthermore, triplet combination treatments of PD-L1 inhibition with PARPi and VEGF inhibitors are currently on the way. For instance, the doublet or triplet combination of durvalumab with olaparib and the VEGFR inhibitor cediranib is the focus of a phase 1/2 study (NCT02484404) in advanced or recurrent solid cancer. Preliminary results show that the recommended dose was tolerable and yielded a 67% clinical benefit rate in nine women with pretreated recurrent solid tumors of which 1 TNBC (141). Results from the MEDIOLA (NCT02734004) clinical trial are pending. This open basket study aims to compare the safety and efficacy of durvalumab in combination with the PARPi olaparib or in combination with olaparib plus the VEGF inhibitor bevacizumab in patients with advanced solid tumors including *BRCA1/2*-deficient breast cancer. Furthermore, it would be of interest to study the clinical benefit of combining PARPi, PD1/PD-L1 blockade and cyclin dependent kinase (CDK) inhibitors. Cyclin dependent kinases are well known master regulators of cell cycle progression and DNA repair pathways, and CDK inhibitors have been shown to sensitize breast cancer cells to PARPi which may further augment the treatment response to immune checkpoint blockade (145). Furthermore, CDK4/6 inhibitors have been found to promote anti-tumor immunity through the stimulation of effector T cell activity, inhibition of proliferation of immunosuppressive regulatory T cells, induction of fibroblast-derived pro-inflammatory cytokines and increased cell surface antigen presentation (146, 147). Another strategy to combine immune checkpoint blockade with targeted therapy involves the inhibition of the MAPK pathway, which is often dysregulated in TNBC and is associated with increased cell proliferation and resistance to apoptosis (148). The phase 2 COLET (NCT02322814) study evaluated the added benefit of combining the MEK1/2 inhibitor cobimetinib with atezolizumab and paclitaxel/nab-paclitaxel as first line treatment in locally advanced or metastatic TNBC. Interim analysis reveals an ORR of 34% in combination with paclitaxel and 29% with nab-paclitaxel (142). In addition, clinical trials using the MEK inhibitor binimetinib in combination with pembrolizumab (NCT03106415) or avelumab (InCITe/NCT03971409) in locally advanced or metastatic TNBC are currently ongoing.

**Table 3:** PD1/PD-L1 antibody-targeted therapy combinations in locally advanced or metastatic TNBC.

NCT Number	Other IDs	Intervention	Trial status/interim results	Ref
**NCT02657889**	TOPACIO/KEYNOTE-162	pembrolizumab + niraparib	active ORR 29% (67%^*^)	(140)
**NCT03167619**	DORA, 3000-PN162-01-001	durvalumab + olaparib	recruiting	
**NCT03801369**	STUDY00018504, NCI-2019-00388, STUDY00018504	durvalumab + olaparib	recruiting	
**NCT02849496**	NCI-2016-01130, 1608018258, 10020, UM1CA186644/86/88/89/91, UM1CA186709	atezolizumab + olaparib	recruiting	
**NCT02484404**	150145, 15-C-0145	durvalumab + olaparib + VEGFRi	recruiting	
**NCT02734004**	MEDIOLA, D081KC00001, 2015-004005016	durvalumab + olaparib +/- VEGFRi	active	(141)
**NCT02322814**	COLET, WO29479, 2014-002230-32	atezolizumab + taxanes + MEKi	active ORR 29–34%	(142)
**NCT03106415**	MC1632, NCI-2017-00496, P30CA015083	pembrolizumab + MEKi	recruiting	
**NCT03971409**	187519, NCI-2019-01531, TBCRC 047, BRE16-279,	avelumab + MEKi	recruiting	

MEKi, MEK inhibitors; ORR, overall response; VEGFRi, VEGFR inhibitors; ^*^BRCA mutant.

### PD1/PD-L1 Antibody-Vaccine Combination Treatment

The use of peptide vaccines for the treatment of metastatic cancer patients has been challenged by low response rates, however, using a multi-peptide vaccine approach the response rates have increased to 9.9% in different cancer types (149, 150). Moreover, combining cancer vaccines with immune checkpoint inhibitors may enhance the anti-tumor immune response elicited by the vaccine. The current clinical trials using PD/PD-L1 antibody-vaccine combination treatments are summarized in **Table 4**. Few ongoing trials are investigating the efficacy of combining cancer vaccines with pembrolizumab, using either the multi-peptide vaccine PVX-410 (NCT03362060), or specific vaccine targeting p53 (NCT02432963) or WT1 (NCT03761914) in advanced TNBC. Additionally, there are few clinical trials exploring the efficacy of combining durvalumab with the multi-peptide vaccine PVX-410 (NCT02826434) or with a neoantigen vaccine (NCT03199040, NCT03606967), and of atezolizumab with a neoantigen vaccine (NCT03289962).

**Table 4:** PD1/PD-L1 antibody-vaccine combination treatment in locally advanced or metastatic TNBC.

NCT Number	Other IDs	Intervention	Trial status
**NCT03362060**	17-328	pembrolizumab + PVX-410	recruiting
**NCT02432963**	15002, NCI-2015-00653	pembrolizumab + p53-specific vaccine	active
**NCT03761914**	SLS17-201/MK3475-770	pembrolizumab + WT1-specific vaccine	recruiting
**NCT02826434**	16-132	durvalumab + PVX-410	active
**NCT03199040**	201710109, 1R01CA240983-01	durvalumab + neoantigen DNA vaccine	recruiting
**NCT03606967**	NCI-2018-01581, 10146, UM1CA186704	durvalumab + Nab-paclitaxel+ neoantigen vaccine	unknown
**NCT03289962**	GO39733, 2017-001475-23	atezolizumab + neoantigen vaccine	recruiting

PVX-410, multi-peptide vaccine (XBP1 US_184-192_; XBP1 SP_367-375_; CD138_260-268_; and CS1_239-247_).

### PD1/PD-L1 Antibody-Natural Killer Cell Combination Treatment

Natural killer (NK) cells form the first line natural defense against abnormal cells and infection with a wide range of pathogens. However, tumor cells have found ways to escape NK cell-mediated immunosurveillance such as the shedding of stress-inducible ligands MHC class I polypeptide–related sequence A (MICA) and MICB, which are exclusively expressed in stressed or transformed cells (151, 152). This results in downregulation of the activating Natural killer group 2 member D (NKG2D) receptor and reduced susceptibility to NK cytotoxicity due to reduced cell surface density of the ligands. NK-based immunotherapy studies are investigating the use of vast numbers of *ex vivo* expanded autologous NK cells, strategies to boost NK cell activity or target inhibitory NK receptors, and the development of genetically engineered NK cells to overcome the immunosuppressive environment (153–155). NK-based immunotherapy in combination with PD-1/PD-L1 immune checkpoint blockade is relatively less studied, with only two clinical trials in TNBC as shown in **Table 5**. The combination of avelumab with iPSC-derived NK cells (FT-516) expressing a high-affinity, non-cleavable variant of the NK activating receptor CD16 (hnCD16) is currently under investigation in multiple advanced solid cancers, including TNBC (NCT04551885). Furthermore, the ongoing landmark trial QUILT-3.067 (NCT03387085) evaluates the safety and efficacy of NK cell combination immunotherapy in patients with refractory, metastatic or unresectable TNBC tumors. The study is unique in design as it combines the use of immune checkpoint inhibition (avelumab) with high-affinity NK (haNK) cell therapy, IL-15 cytokine administration, cancer vaccines and metronomic chemoradiation to stimulate both the innate and adaptive immune system. Interim results of nine patients demonstrate an overall response rate of 67% with a disease control response rate of 78% and complete response rate of 22% (156). Notably, the duration of the treatment responses with a median PFS of 13.7 months is very promising in comparison to the historical PFS of 3 months.

**Table 5:** PD1/PD-L1 antibody-NK cell combination treatment in advanced or metastatic TNBC.

NCT Number	Other IDs	Intervention	Trial status	Ref
**NCT04551885**	FT516-102	Avelumab + FT-516	Recruiting	
**NCT03387085**	QUILT-3.067	Avelumab + haNK + IL-15 + vaccine + chemoradiation	Active ORR 67% PFS (13.7 mths)	(156)

FT-516, iPSC-derived NK cells with hnCD16; IL-15, interleukin 15; NK, natural killer cell; ORR, overall response rate; PFS, progression free survival.

## Predictive Biomarkers in Immune Checkpoint Inhibition

Immune checkpoint blockade has entered clinical practice as first- or second-line treatment for a number of cancers, however, it remains a challenge to select patients that will benefit the most. PD-L1 expression is widely used as predictive biomarker due to its association with better response rates to PD1/PD-L1 blockade for patients with mTNBC. As described above, stronger PD-L1 positivity has been associated with better overall response rates, progression-free, and overall survival in metastatic TNBC patients treated with ICI monotherapy or in some cases with a chemotherapy combination (40, 124, 129, 134). Routine clinical testing of PD-L1 expression is currently conducted using five distinct FDA-approved companion diagnostic immunohistochemistry tests (157). Nevertheless, the use of different antibody clones (22C3 for pembrolizumab, 28-8 for nivolumab, SP263 for durvalumab, SP142 for atezolizumab, and 73-10 for avelumab), biomarker staining platforms, scoring systems and cut-off values for PD-L1 positivity makes it very difficult to consolidate the predictive value of PD-L1 expression across tumor types and across studies. Moreover, some assays define PD-L1 positivity solely based on tumor cell surface expression while others quantify cytoplasmic plus cell surface PD-L1 expression of tumors and immune cells. The prospective multi-institutional Blueprint study compared the performance of all five PD-L1 antibody clones in non-small cell lung cancer specimens (158). They reported good concordance among three antibodies (22C3, 28-8, and SP263), while the fourth antibody clone (73-10) demonstrated superior sensitivity and the fifth clone (SP142) underperformed with lower sensitivity. Similarly, high concordance has been reported between clones 22C3, 28-8, and SP142 in primary and metastatic urothelial carcinomas with the lowest sensitivity again being associated with SP142 (159). PD-L1 scoring of head and neck squamous cell carcinoma, urothelial carcinoma and breast cancer revealed a higher inter-observer variability for clone SP142 as compared to clones SP263 and 22C3 (160). In TNBC, few studies compared the performance of the FDA-approved assays and corroborated the previous findings in which SP142 detected significant less PD-L1 positivity compared to SP263 and 22C3 (161–163). A recent study involved 19 pathologists from 14 different institutions to evaluate the sensitivity and reproducibility of SP142 and SP263 staining in advanced TNBC (164). This study reported PD-L1 positivity in 58% of cases using SP142 and in 78% with SP263, with decreased observer agreement of 41% at eight observers for SP142 and 46% at 10 observers for SP263. Despite the lower performance of SP142, the SP142-based Ventana test currently remains the companion diagnostic test for the first FDA-approved immunotherapy regimen of atezolizumab plus nab-paclitaxel treatment of patients with metastatic, locally advanced or unresectable tumors, based on the results from the Impassion130 trial (128, 135). Of note, soluble PD-L1 (sPD-L1) has been detected in the peripheral blood of patients with advanced non-small cell lung cancer, multiple myeloma, diffuse large B-cell lymphoma, and renal cell carcinoma whereby high levels are associated with poor prognosis (165–168). High pre-treatment sPD-L1 levels were associated with worse outcome in melanoma patients treated with ipilimumab or pembrolizumab, which could possibly reflect a larger tumor burden and/or an exhausted immune response that cannot be reinvigorated by immune checkpoint blockade (168). In contrast, an increase in post-treatment sPD-L1 was associated with partial response. These findings highlight the need for less ambiguous, more reproducible predictive biomarkers for immune checkpoint inhibition.

Two emerging predictive biomarkers are the number of tumor infiltrating lymphocytes and the tumor mutational burden. Increased number of TILs have been associated with better overall survival in TNBC patients treated with ICI monotherapy or in combination with chemotherapy (117, 136). The relative importance of intratumoral TILs (iTILs) versus stromal TILs (sTILs) has not clearly been defined yet and might differ between tumor types. In breast cancer, both iTILs and sTILs have been correlated with clinical outcome and chemotherapy response (59, 60, 63, 78, 169). Moreover, in metastatic TNBC sTILs have been correlated with treatment response to pembrolizumab, atezolizumab, and nivolumab (117, 124). Thus, the International Immuno-Oncology Biomarker Working Group published guidelines for the assessment of stromal and intra-tumoral TILs in a wide range of solid tumor types (170). However, robust scoring of sTILs is hindered by differences in relative iTIL and sTIL distribution, inaccurate delineation of tumor boundaries, small areas of intratumoral stroma, presence of necrosis and extracellular mucin (171). Furthermore, tumor mutational burden has been correlated with higher objective response rates to anti-PD1 or anti-PD-L1 monotherapy across 27 solid tumor types (29). Interestingly, in breast cancer lower response rates were observed than expected based on TMB suggesting that TMB might not be a good predictive biomarker in these tumors. We believe that a combination of predictive biomarkers such as PD-L1 expression, iTIL and sTIL density together with TMB, TCR diversity and immune gene signatures will more likely yield improved performance over each of these biomarkers alone, therefore warranting further investigation.

## Conclusion

To conclude, the results of immune checkpoint blockade clinical trials in TNBC are promising, in particular in metastatic setting. The FDA-approval of atezolizumab plus nab-paclitaxel for metastatic TNBC marks the first licensed immunotherapy regimen for breast cancer. Combining immune checkpoint inhibition with chemotherapy, PARP inhibitors, cancer vaccines or NK cell therapy holds great potential to increase the clinical benefit in TNBC. Nevertheless, we highlight here that the selection of patients with the highest likelihood of benefit from these treatments requires reliable predictive biomarkers as well as a better understanding of cancer cell-intrinsic and/or microenvironmental factors that define a potent and durable anti-tumor immune response.

## Author Contributions

RT drafted the manuscript. GA-K designed the figure and tables and critically revised the manuscript. JD conceived and critically revised the manuscript and tables. All authors contributed to the article and approved the submitted version.

## Funding

This work was supported by grants from the Qatar Biomedical Research Institute (#VR94), awarded to JD, and the Qatar National Research Fund (QRLP10-G-1803024), awarded to GA-K.

## Conflict of Interest

The authors declare that the research was conducted in the absence of any commercial or financial relationships that could be construed as a potential conflict of interest.
